# Enhanced vitamin B_12_ production by isolated Bacillus strains with the application of response surface methodology

**DOI:** 10.1186/s12896-024-00919-5

**Published:** 2024-11-12

**Authors:** Rania M. M. Abdel-Baki, Marwa N. Ahmed, Olfat S. Barakat, Galal M. Khalafalla

**Affiliations:** Department of Microbiology, Faculty of Agriculture, Cairo University, El-Gamaa Street, Giza, 12613 Egypt

**Keywords:** Vitamin B_12_, Cyanocobalamin, Methylcobalamin, *Peribacillus acanthi*, Molasses, Plackett–Burman design

## Abstract

**Background:**

Vitamin B_12_ is a crucial B-group vitamin, first isolated from the liver due to its role in combating pernicious anemia. It is distinguished by its unique and complex structure, which makes its chemical synthesis challenging and expensive. Consequently, vitamin B_12_ is alternatively obtained through microbial fermentations. Molasses, an affordable and safe agro-industrial waste, can be used as a carbon source for vitamin B_12_ production, offering a cost-effective alternative to expensive sugars in the production medium.

**Results:**

A total of 87 yeast, actinomycete, and bacterial isolates were screened for vitamin B_12_ production, with 15 isolates showing high productivity. *Bacillus* isolates were selected for further analysis using MALDI-TOF and molecular identification. These isolates were identified as four strains of *Bacillus subtilis* (MZ08, JT10, BY11, and JT17), one strains of *Bacillus sp.* (CB09), and one strain of *Peribacillus acanthi* (MZ01). Genetic circuits associated with vitamin B_12_ production were demonstrated in a closely related strain of *Peribacillus acanthi* MZ01 strain*.* Three strains (MZ01, MZ08, and JT17) were selected for further evaluation of vitamin B_12_ productivity under different sugar types (glucose, sucrose, fructose, lactose, and galactose) and varying inoculum sizes. The inoculum size significantly impacted vitamin B_12_ production, with an increase from 5 to 10% enhancing yields. The ability of the strains to produce vitamin B_12_ varied depending on the type of sugar used. *Peribacillus acanthi* MZ01 strain showed the highest productivity and subsequently, selected for optimizing vitamin B_12_ production conditions using response surface methodology. Furthermore, the optimized conditions were then applied to molasses-based medium to achieve high vitamin B_12_ yields by MZ01 strain.

**Conclusion:**

In this study, *Peribacillus acanthi* was characterized for the first time as a vitamin B_12_ producer, demonstrating high productivity among various tested strains. The optimization of production conditions using response surface methodology, further enhanced vitamin B_12_ yields, showcasing the strain’s efficiency in microbial fermentations. This research also highlights the potential of using molasses as a cost-effective alternative carbon source, significantly reducing production costs.

**Supplementary Information:**

The online version contains supplementary material available at 10.1186/s12896-024-00919-5.

## Introduction

Vitamin B_12_, also known as cobalamin, is a crucial water-soluble vitamin involved in various metabolic processes. It acts as a cofactor in the metabolism of lipids, carbohydrates, amino acids, and nucleic acids [1]. This vitamin is essential for the formation of red blood cells, supports the brain and nervous system, and plays a vital role in DNA synthesis [2]. Deficiency in vitamin B_12_ can lead to significant health issues, including megaloblastic anemia, neurological disorders, and cognitive decline [3]. Vitamin B_12_ was first isolated in its cyanoform as an anti-pernicious anemia factor from liver extracts in 1948 [4]. Its main dietary sources are animal-based foods such as red meat, milk, cheese, eggs, fish, and shellfish [5]. Due to the lack of plant-based sources, vegans and vegetarians are particularly at risk of vitamin B_12_ deficiency, which can manifest in neurological symptoms, dementia, psychiatric disorders, and pernicious anemia [6]. Vitamin B_12_ exists in four forms: methylcobalamin, deoxyadenosylcobalamin, hydroxocobalamin, and cyanocobalamin [7]. Among these, methylcobalamin (MeCbl) and deoxyadenosylcobalamin (AdoCbl) are biologically active forms that function as coenzymes. However, these active forms are sensitive to light and can degrade into hydroxocobalamin at ambient temperatures in aqueous solutions. To enhance stability and shelf life, most commercial vitamin B_12_ supplements are produced as cyanocobalamin (CNCbl), a more stable form that can be converted into the active coenzymes within the human body [8].

The industrial production of vitamin B_12_ can be accomplished through both chemical synthesis and microbial fermentation. [9]. However, chemical synthesis poses challenges due to its complexity, high costs, and environmental impact. Consequently, microbial fermentation has become the preferred method for large-scale production. *Propionibacterium freudenreichii* have been widely used in industrial settings for vitamin B_12_ production [10]. Additionally, lactic acid bacteria such as *Lactobacillus* spp. and *Lactococcus* spp. have shown potential for vitamin B_12_ production [11]. Recent studies have also explored the use of *Mesorhizobium loti* and *Bacillus megaterium* for vitamin B_12_ production [12, 13]. To further enhance the cost-effectiveness and sustainability of vitamin B_12_ production, the use of inexpensive and readily available raw materials, such as molasses, has been explored. Molasses, a byproduct of the sugar refining industry, is rich in sugars (sucrose, glucose, and fructose) and other nutrients that can support microbial growth. Utilizing molasses as a fermentation substrate offers several advantages, including reduced production costs and the recycling of industrial byproducts [14]. Microorganisms such as *Propionibacterium freudenreichii* and *Bacillus megaterium* have demonstrated the ability to produce high yields of vitamin B_12_ using molasses-based media, highlighting its potential as a sustainable feedstock [15].

Therefore, this study aims to optimize the production of vitamin B_12_ through microbial fermentation using *Bacillus* spp. strains isolated from marine and food sources in Egypt. Furthermore, this research aims to to maximize the yield of this essential vitamin under a range of fermentation conditions utilizing molasses as a cost-effective substrate and applying Response Surface Methodology (RSM) for optimization. RSM is a statistical and mathematical technique that helps in identifying the optimal conditions for multivariable systems by evaluating the interactions between different parameters [16]. RSM has been used previously to optimize various fermentation parameters such as temperature, pH, substrate concentration, and nutrient supplementation for enhancing vitamin B_12_ production [17, 18].

The findings of this research have the potential to advance the sustainable and cost-effective production of vitamin B_12_ at an industrial scale by leveraging *Bacillus* spp. strains isolated from different environmental and food sources in Egypt.

## Materials and methods

### Sampling

Marine water samples were obtained from El-Ein El-Sokhna, located along the Red Sea coast in Egypt. Lake water samples were collected from Ein Al-Sira Lake in Cairo, Egypt. Corn and Juhayna Rayeb samples were collected from multiple markets within the Giza city. Soil samples were collected from the El-Manial district in Cairo, Egypt.

### Raw material

Egyptian sugarcane molasses was obtained from El-Hawamdia factory for the integrated sugar industry and clarified to be implemented as an agricultural waste material for vitamin B_12_ production via microorganisms being isolated in this study.

### Bacterial strains

The strain *Lactobacillus leichmannii* ATCC 7830 was utilized as both a test and indicator organism for the production of vitamin B_12_. This strain was obtained from the Microbial Resource Center at the Faculty of Agriculture, Ain Shams University, Cairo, Egypt.

### Isolation and screening of vitamin B_12_ producing isolates

Bacterial isolation from marine water and food samples was performed using nutrient agar medium, following the pour plate technique, and incubating at 30ºC for 24–48 h [19]. The isolates obtained were then screened for vitamin B_12_ production by cultivating them on vitamin B_12_ assay medium at 30ºC for 24–48 h, as described previously [1, 20]. The ability of the isolates to grow on this medium in the absence of exogenous vitamin B_12_ indicated their potential to synthesize the vitamin.

To further verify the vitamin B_12_ production capabilities of the isolates, an auxotrophic indicator strain, *Lactobacillus leichmannii* ATCC 7830, was employed. This strain can only grow in the presence of vitamin B_12_. The assay involved plating the strain on vitamin B_12_-free agar medium, where two wells were created in each plate using a sterile cork borer. Each well was filled with 100 μl of the supernatant from the selected isolates suspected to produce vitamin B_12_. For control, 100 μl of a standard cyanocobalamin solution (2–10 μg/mL, LOBA CHEMIE PVT. LTD) was added to two wells per plate. The plates were then incubated at 37°C for 24 h and examined for growth zones around the wells, indicating vitamin B_12_ production [21, 22].

*Lactobacillus leichmannii* ATCC 7830 was cultured and maintained on MRS Broth (TM MEDIA) at 37ºC for 24 h. The cultures were then centrifuged, washed with saline, and resuspended in 10 mL of saline to serve as the indicator organism for vitamin B_12_ production.

The isolates confirmed as vitamin B_12_ producers were subjected to morphological characterization using the Gram stain method and preserved as pure cultures at -80ºC for further analysis.

### Vitamin B_12_ production

#### Inoculum Preparation

The inoculum was prepared by inoculating the selected *Bacillus* isolates into Erlenmeyer flasks containing 100 mL of sterile inoculum medium with the following composition (g/L): peptone 5, yeast extract 3, glucose 10, potassium dihydrogen phosphate 2, and Tween-80 0.1 [23]. The cultures were incubated at 30°C for 24 h in a shaking incubator (Innova 4300) set at 110 rpm.

##### Fermentation

Five milliliters of the prepared inoculum were introduced into 100 mL of Zaky’s production medium, which contained the following (g/100 mL): xylose 12, glucose 12, malt extract 1.2, yeast extract 1.2, peptone 2, (NH_4_)_2_SO_4_ 0.4, KH_2_PO_4_ 0.025, and CoSO_4_·7H_2_O 1 [24]. The mixture was fermented at 30°C for 2 days using a shaking  incubator (Innova 4300) set at 110 rpm. After fermentation, the dry biomass of the vitamin B_12_-producing isolates, and the yield of vitamin B_12_ were measured.

##### Biomass estimation

Biomass was estimated by centrifuging 100 mL of the isolate cultures at 10,000 rpm for 10 min using a HERMLE Z323K centrifuge. The resulting pellet was then dried to a constant weight at 103–105°C in a vacuum oven [15].

##### Extraction and estimation of Vitamin B_12_

Vitamin B_12_ extraction was carried out by first harvesting the cells from the fermentation broth through centrifugation at 17,000 rpm for 15 min [15]. The resulting pellet was washed, and one gram of the sample was decomposed in 50 mL of sterile buffer solution (pH 6) containing (g/100 mL distilled water): disodium hydrogen phosphate 1.29, citric acid 1.1, sodium metabisulfite 1, and potassium cyanide 0.01. The mixture was then autoclaved at 121°C for 15 min to denature the proteins. The supernatant, containing the extracted vitamin B_12_, was centrifuged to obtain a particle-free solution. The vitamin B_12_ concentration in the extracted solution was then measured spectrophotometrically at 560 nm using a PG Instruments T60 UV–Vis spectrophotometer [25].

For HPLC analysis, One gram of the cell pellet was then suspended in 50 mL of a sterile phosphate-citrate buffer (pH 6.0) containing the following components (g/100 mL distilled water): disodium hydrogen phosphate 1.29 g, citric acid 1.1 g, sodium metabisulfite 1 g, and potassium cyanide 0.01 g.

This mixture was autoclaved at 121°C for 15 min to denature the cellular proteins and release the intracellular vitamin B complex. Post-autoclaving, the suspension was centrifuged at 12,000 rpm for 10 min to obtain a clear, particle-free supernatant.

### HPLC analysis

The HPLC analysis was conducted using a Thermo (Ultimate 3000) system, which included an automatic sample injector, a DAD-3000 diode array detector, and a DELL-compatible computer running Chromelion7 software for data interpretation. The mobile phase, standard solutions, and a 0.45 µm Millipore membrane filter were degassed and filtered prior to analysis. Compound identification was achieved by comparing their UV absorption spectra and retention times with those of the standard.

A purified 20 µL sample was injected onto an SVEA-RP-C18 gold Inertsil column (250 mm × 4.6 mm, 5 µm) at 25°C. The mobile phase consisted of methanol (55:35:10, v/v) and KH_2_PO_4_ (25 mM) with the pH adjusted to 3.8 using phosphoric acid, and the flow rate was set at 1.0 mL/min. Detection was carried out at 220 nm [26].

### Identification of vitamin B_12_ producing isolates using MALDI-TOF MS analysis

Selected vitamin B_12_-producing isolates were analyzed using MALDI-TOF MS as described previously [27, 28]. Briefly, fresh 24–48 h cultures were subjected to full protein extraction by mixing with 50 µL of sterile water and 50 µL of 96% ethanol. One microliter of the suspension was then spotted onto a 96-spot stainless steel target plate (Bruker Daltonics, Bremen, Germany) and allowed to air dry for 2 min. The dried spots were overlaid with 1 µL of 70% formic acid, followed by 1 µL of HCCA matrix (Bruker Daltonics). Once fully dried, the plates were analyzed by MALDI-TOF MS. The resulting mass spectra were compared to a reference database or library, yielding a list of the closest matching isolates, ranked numerically based on similarity.

### Molecular identification of the potential vitamin B_12_ producing isolate

The bacterial strain exhibited a high vitamin B_12_ production was identified by amplifying and sequencing the 16S rRNA gene using universal forward (5′-AGAGTT TGATCMTGGCTCAG-3′) and reverse (5′-AAGGAGGTGATCCAGCC-3′) primers. PCR reactions were carried out following a previously established protocol. The resulting PCR products were visualized using gel electrophoresis and a gel documentation system (Bio-Rad, USA), followed by purification and sequencing. Alignment of the 16S rRNA sequences with bacterial sequences in the NCBI database was performed using the basic local alignment search tool (BLAST). Phylogenetic analysis was conducted using Molecular Evolutionary Genetics Analysis Version 11 (MEGA 11), employing the Muscle algorithm for aligning the 16S rRNA sequences (Gap open: -400, gap extend: 0, and UPGMA clustering) [29]. The Maximum Likelihood (ML) method was employed to construct the 16S rRNA tree. Evolutionary distances were calculated using the Kimura 2-parameter method [30], and the robustness of the phylogenetic relationships was assessed through 1000 bootstrap replicates.

### In silico analysis of vitamin B_12_ biosynthetic gene clusters

The PATRIC platform, the all-bacterial Bioinformatics Database and Analysis Resource Center available online at www.bv-brc.org, was implemented to unravel and visualize the vitamin B_12_ biosynthetic gene clusters in *Peribacillus acanthi* L28 strain. This analysis involved comparing these clusters across analogous gene regions in other bacterial strains known for their vitamin B_12_-producing capabilities. Additionally, the Uniprot online platform was utilized to annotate the functions of genes implicated in the vitamin B_12_ production pathways (The UniProt Consortium 2023).

#### Pathogenicity and hemolysis of identified strains

The hemolysis test on blood agar plates was performed following the protocol described by Buxton (2005) [31] to assess the pathogenicity and hemolytic properties of the identified strains. The selected isolates were streaked onto blood agar plates and incubated at 30°C for 24 h. Isolates exhibiting gamma (γ) or no hemolysis were classified as safe or non-pathogenic.

#### One Factor At-A-Time (OFAT) optimization of vitamin B_12_ production

Strains that exhibited no hemolysis and demonstrated high vitamin B_12_ productivity were selected for optimization to determine the ideal conditions for their production. Optimization was performed by varying one factor at a time while keeping other factors constant. A total of eight factors were studied, with two factors chosen for variation: sugar type (glucose, sucrose, fructose, lactose, and galactose) and inoculum size (5%, 10%, and 15%). The constant conditions were temperature (30ºC), fermentation time (2 days), salt concentration (0%), pH (6.5), sugar concentration (12%), and shaking rate (110 rpm).

#### Optimization of vitamin B_12_ production by statistical modeling using response surface methodology (RSM)

The most promising MZ01 strain that can produce the highest yields of vitamin B_12_ was selected for optimization using response surface methodology (RSM) by the Placket-Burman design.

Placket-Burman design was applied to design fermentation experiments as a modeling technique. This design was implemented to assess the relationships between set of variables and observed results. Twenty-nine fermentation runs were designed based on six factors-temperature, A (ºC); fermentation time, B (days); salt concentration, C (%); pH, D; glucose concentration, E (%); shaking rate, F (rpm). Each variable was coded at three levels (− 1, 0, + 1) to describe the most optimum response surface (Table 1). The responses produced from this experimental design were the vitamin B_12_ concentration and the biomass yield.

**Table 1:** The experimental design matrix of the variables involved in the modeling using RSM

**Run**	**Std**	**A**	**B**	**C**	**D**	**E**	**F**
**1**	18	30.5	60	2	6.5	12	100
**2**	26	30	60	2.2	6.5	12	100
**3**	4	35	96	4	5.5	6	200
**4**	20	30	60	2	6.6	12	100
**5**	2	35	24	4	7.5	6	200
**6**	11	25	96	0	7.5	6	0
**7**	19	30	60	2	6.4	12	100
**8**	22	30	60	2	6.5	12	110
**9**	21	30	60	2	6.5	12	90
**10**	5	25	96	4	7.5	18	0
**11**	28	30	63.6	2	6.5	12	100
**12**	17	29.5	60	2	6.5	12	100
**13**	27	30	56.4	2	6.5	12	100
**14**	8	35	96	4	7.5	6	0
**15**	14	25	96	4	5.5	18	200
**16**	24	30	60	2	6.5	12.6	100
**17**	23	30	60	2	6.5	11.4	100
**18**	25	30	60	1.8	6.5	12	100
**19**	16	25	24	0	5.5	6	0
**20**	13	25	24	4	5.5	18	0
**21**	7	25	24	0	7.5	6	200
**22**	9	35	24	0	5.5	18	0
**23**	29	30	60	2	6.5	12	100
**24**	6	35	96	0	5.5	18	200
**25**	12	35	24	4	5.5	6	0
**26**	1	35	24	0	7.5	18	200
**27**	15	25	24	4	7.5	18	200
**28**	10	25	96	0	5.5	6	200
**29**	3	30	96	0	7.5	18	0
**Symbol**	**Factor**				**Levels**		
					**-1**	**0**	**1**
**A**	Temperature (ºC)				25	30	35
**B**	Fermentation time (hours)				24	60	96
**C**	Salt concentration %				0	2	4
**D**	pH				5.5	6.5	7.5
**E**	Glucose concentration %				6	12	18
**F**	Shaking rate (rpm)				0 (static)	100	200

#### Production of vitamin B_12_ using synthetic molasses medium

Clarified molasses was selected as a waste material to serve as the primary sugar source in the production medium, replacing glucose. The used molasses contained 9.5% glucose, 31% sucrose, 10% fructose, 0.95% nitrogen, and 80% total solids.

For molasses clarification, concentrated H_2_SO_4_ (3 mL) was added to 1 kg of molasses mixed with 1 L of distilled water, and the pH was adjusted to 3.5. The mixture was then boiled for 30 min in a water bath, cooled, and kept in the refrigerator overnight. Afterward, it was centrifuged and sterilized at 121ºC for 15 min. The final sugar concentration of the clarified molasses was 25% [32] (Sadik and Halema, 2014).

Based on the statistical modeling results, the sugar concentration of the clarified molasses was adjusted to 6% and 12%, and the pH was set to 6.5 and 7.5 to maximize vitamin B_12_ production. Consequently, the glucose levels in the production medium and the sugar concentration of clarified molasses were adjusted and optimized according to the production conditions (Table S1).

A synthetic molasses medium was prepared to include all components of the production medium as previously described [33] with glucose replaced by clarified molasses.

#### Statistical analysis

GraphPad prism 10 software and R (version 3.2.5) were used for the statistical analysis to calculate the standard error of the mean and the *p*-value of validation data. The statistical analysis and modeling were performed using the Design-Expert v7.0.0 software (Stat- Ease, Inc. MN, USA).

## Results and discussion

### Isolation and screening of vitamin B_12_-producing isolates

Eighty-Seven isolates were isolated from various samples and sources and screened for vitamin B_12_ production. The cellular morphologies of the isolates were diverse, including bacilli, cocci, oval shapes, and actinomycetes (Table S2). Among the 87 isolates initially screened for vitamin B_12_ production, 9 bacilli and 6 yeast isolates were able to grow on a vitamin B_12_-free agar medium. These isolates also exhibited a growth zone around the wells pre-inoculated with the indicator test organism *Lactobacillus leichmannii* ATCC 7830, indicating they are vitamin B_12_ producers.

The biomass and vitamin B_12_ amount were estimated in each vitamin B_12_-producing isolate.

Table 2 shows the isolates codes, the amount of biomass grown, and the vitamin B_12_ yield. *Bacillus* isolates MZ01, MZ08, JT10, and CB09 produced the highest vitamin B_12_ yields, with concentrations of 174.14, 174.14, 173.91, and 141.36 µg/100 mL of culture, respectively. In contrast, *Bacillus* isolates JT17, LT13, and LT12 produced significantly lower vitamin B_12_ yields, with concentrations of 10.60 ± 0.03, 5.35 ± 0.01, and 0.89 ± 0.00 µg/100 mL of culture, respectively.

**Table 2:** Biomass and vitamin B_12_ yield produced by the selected bacilli isolates

Isolate code	Source	Biomass g/100 mL	Vitamin B_12_µg/100 mL culture	Vitamin B_12_µg g^−1^cells culture
**MZ01**	Marine	0.9 ± 0.02	174.14 ± 0.11	193.49
**MZ08**	Marine	0.4 ± 0.01	174.14 ± 0.13	435.35
**CB09**	Corn	0.1 ± 0.00	141.36 ± 0.11	1413.6
**JT10**	Rayeb	0.1 ± 0.00	173.91 ± 0.11	1739.1
**BY11**	Soil	0.2 ± 0.01	95.65 ± 0.07	478.25
**LT12**	Lake	0.09 ± 0.00	0.89 ± 0.00	9.89
**LT13**	Lake	0.46 ± 0.02	5.35 ± 0.01	11.63
**MZ14**	Marine	0.6 ± 0.02	9.59 ± 0.01	15.98
**JT17**	Juhayna Rayeb	0.1 ± 0.00	10.60 ± 0.03	106

Production conditions: 12% glucose as a type of sugar, 10% inoculum size, 30°C, and aeration rate of 110 rpm

Sitoresmi et al. (2021) revealed that the strain *Lysinibacillus fusiformis*, isolated from Ranu Grati Lake, produced the highest vitamin B_12_ yield (33,783 µg/mL) among the tested strains in their study [34]. This highlights the potential of environmental *Bacillus*-related strains, including those from various habitats like lakes, to serve as prolific producers of vitamin B_12_. Moreover, *Bacillus megaterium* and many *Bacillus* species have been demonstrated to be potent vitamin B_12_ producers [35, 36]. These studies establish the relevance of *Bacillus* species as valuable producers of vitamin B_12_.

### HPLC analysis

**Fig. 1:**
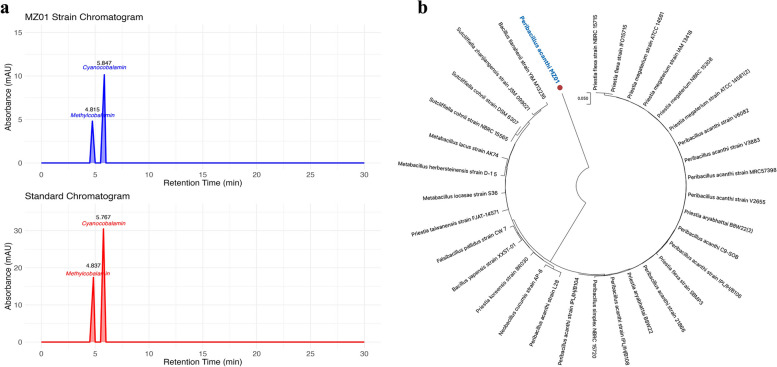
**a** HPLC chromatogram of vitamin B_12_ showing peaks for MZ01 strain and standard. Vitamin B_12_ detected as cyanocobalamin and methylcobalamin. **b** The phylogenetic tree of the *Peribacillus acanthi* MZ01 strain isolated and sequenced in this study, showing their 16S rRNA sequences in relation to the closest strains

Vitamin B_12_ exists in four forms: hydroxocobalamin and cyanocobalamin, which are commonly used and must be converted by the body into the metabolically active forms, methylcobalamin and adenosylcobalamin, to support cobalamin-dependent enzyme functions [37]. The MZ01 isolate was analyzed using HPLC to determine the levels of vitamin B_12_ in its cyanocobalamin and methylcobalamin forms (Fig. 1a). The analysis revealed that MZ01 produced a higher yield of cyanocobalamin (200 µg/mL) compared to methylcobalamin (121 µg/mL). Cyanocobalamin was detected with a retention time of 5.847 min, while methylcobalamin had a retention time of 4.815 min.

This finding is consistent with the results of Mohammed et al. (2014), which employed HPLC analysis for the detection of vitamin B_12_. This study demonstrated that *Bacillus megaterium* can produce both adenosylcobalamin and cyanocobalamin forms of vitamin B_12_ [38].

### Identification of the vitamin B_12_-producing isolates using MALDI-TOF MS technique

Six vitamin B_12_-producing *Bacillus* isolates, demonstrating high productivity, were selected for identification using MALDI-TOF MS. One isolate was further subjected to molecular identification. The identity and similarity percentages of the identified strains are shown in Table S3. The results confirmed that isolates MZ01 and CB09 were identified as *Bacillus* species, while MZ08, JT10, BY11, and JT17 were identified as *Bacillus subtilis*, with similarity percentages exceeding 98.7%. These findings were consistent with the morphological characteristics of *Bacillus* isolates. The successful identification of these strains using MALDI-TOF MS aligns with several studies that highlight the technique’s power, sensitivity, and reliability for bacterial identification [39–41].

### Molecular identification of the potential vitamin B_12_ producing isolate

The most promising vitamin B_12_ producing bacterial strain was identified using 16S rRNA sequencing. The obtained sequence was analyzed using the NCBI blast tool and subsequently deposited in NCBI GenBank (https://www.ncbi.nlm.nih.gov/genbank/). The accession number for the bacterial strain is PP301893. Based on the analysis, the bacterial strain was identified as *Peribacillus acanthi* strain (MZ01). Figure 1b illustrates the phylogenetic relationship of the sequenced strain, based on its 16S rRNA sequences, in comparison to the closest bacterial strains.

### In silico analysis of vitamin B_12_ biosynthetic gene clusters

**Fig. 2:**
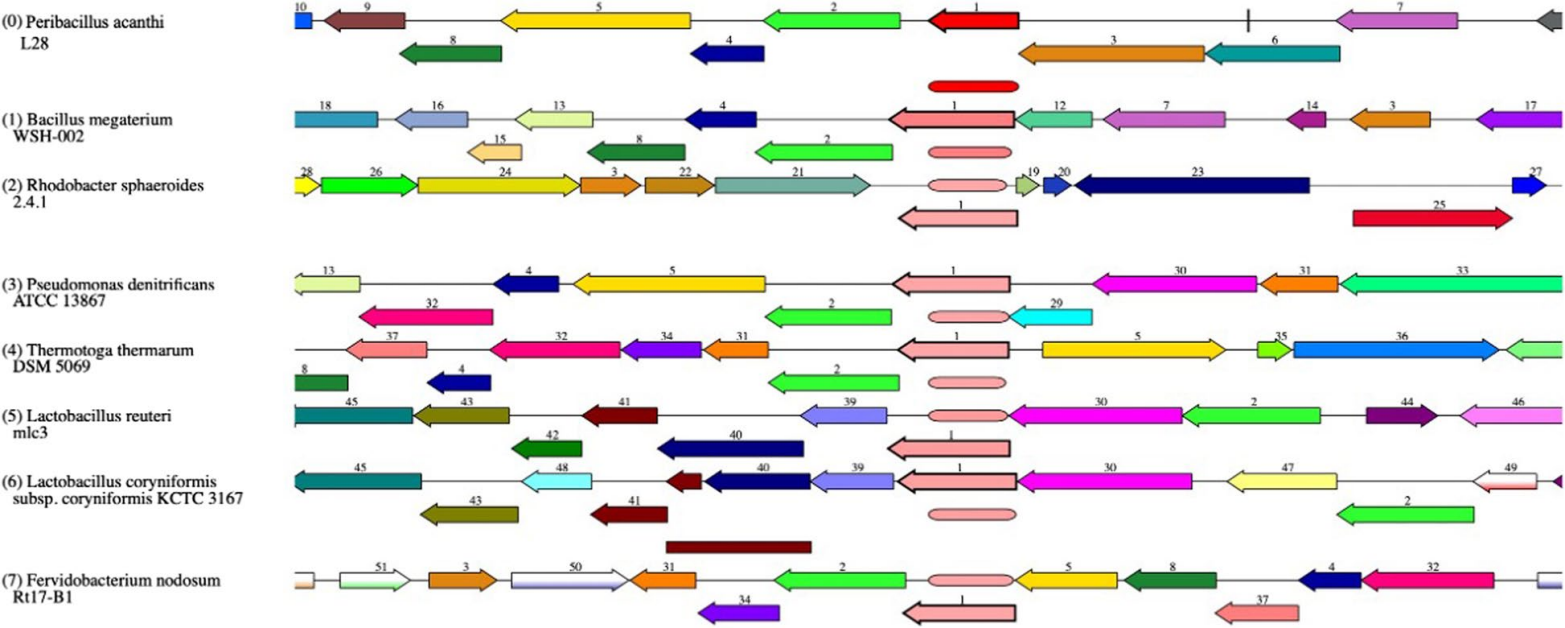
Genetic arrangement of vitamin B_12_ biosynthesis gene cluster in *Peribacillus acanthi* L28 strain in comparison to other vitamin B_12_ producing bacteria. The genes are represented by colorful arrows and numbers: (1-adenosylcobinamide-phosphate synthase *cbiB*, 2-threonine-phosphate decarboxylase *cobD*, 4-adenosylcobinamide kinase *cobU*, 5- cobyric acid synthase *cobQ*, 6-vitamin B_12_ ABC transporter, permease protein *btuC*, 7- vitamin B_12_ ABC transporter, substrate-binding protein *btuF*, 8-cobalamin synthase *cobS*)

The vitamin B_12_ gene clusters within the *Peribacillus acanthi* L28 strain (a previously sequenced strain, available on https://www.ncbi.nlm.nih.gov) [42] were compared with homologous gene regions in other bacterial strains known for vitamin B_12_ production. These clusters exhibit a close genetic relationship with those identified in *Bacillus megaterium* WSH-002 strain (Fig. 2). However, notable differences in gene cluster arrangement were observed in *Peribacillus acanthi* when compared to other bacterial strains. Within the *Peribacillus acanthi* L28 genome, two distinct clusters were identified, designated as Cluster I and Cluster II.

Cluster I encompasses the *cbiBS* genes co-located with *cobDQUS* genes, which are implicated in the later processing steps of cobalamin synthesis. Cluster II harbors *btuCF* genes, pivotal components in the salvage pathway of cobalamin biosynthesis. The *cbi* genes play a role in anaerobic cobalamin synthesis, while the *cob* genes are involved in aerobic cobalamin production. The *btu* genes, crucially, participate in the salvage pathway of cobalamin synthesis, acting as ABC transporters facilitating corrinoid uptake and subsequent delivery to the inner membrane to mediate cobalamin synthesis [43].

These insights indicate the potential of *Peribacillus acanthi* bacterium for vitamin B_12_ production. However, to gain a comprehensive understanding of the vitamin B_12_ biosynthetic circuitry within this bacterium, further complete genome characterization of the *Penibacillus acanthi* MZ01 strain is required.

### Pathogenicity and hemolysis of identified strains

All six identified strains were grown on blood agar media to test their hemolysis. The hemolysis results are listed in (Table S4). All strains showed gamma-hemolysis (non-hemolytic) which indicates their non-pathogenicity nature except CB09 strain showed β hemolysis.

### One Factor At-A-Time (OFAT) optimization of vitamin B_12_ production

Three bacterial strains (MZ01, MZ08, and JT17) were selected for OFAT optimization of vitamin B_12_ production under specific conditions: temperature (30ºC), fermentation time (2 days), salt concentration (0%), pH (6.5), and shaking speed (110 rpm). We explored the effects of varying sugar types (glucose, sucrose, fructose, lactose, and galactose) and inoculum sizes (5%, 10%, and 15%) to determine the optimal conditions for maximizing vitamin B_12_ yield.

According to the data presented in Supplementary Material 2, *Bacillus subtilis* JT17 strain produced the highest vitamin B_12_ yield (15.27 µg/100 mL) when fructose was used as the sugar source (*P* < 0.001), followed by *Peribacillus acanthi* MZ01 strain, which produced 10.03 µg/100 mL using glucose (*P* < 0.001). In contrast, *Bacillus* sp*.* MZ08 strain exhibited the lowest production (1.45 µg/100 mL) when glucose was used. These results suggest that the type of sugar significantly influences vitamin B_12_ biosynthesis. This is consistent with findings by Piwowarek et al. (2018) and Abdel-Baki et al. (2024) showing that the sugar type signifcantly affect the vitamin B_12_ yield [44, 45].

Table 3 shows the impact of inoculum size on vitamin B_12_ production by the *Bacillus* strains. *Bacillus subtilis* JT17 strain consistently increased the vitamin B_12_ yield (8.93, 10.60, 17.88 µg/100 mL) with increasing inoculum sizes (5%, 10%, 15%). In contrast, MZ01 and MZ08 strains showed increased vitamin B_12_ production (*P* < 0.0001) when the inoculum size was incremented from 5 to 10% (yielding 10.03, 174.14 µg/100 mL for MZ01; and 1.45, 174.14, 2.67 µg/100 mL for MZ08). However, increasing the inoculum size from 10 to 15% led to a reduction in vitamin B_12_ yield (*P* < 0.001). Abdel-Baki et al. (2024) showed that inoculum size signifcantly affected the vitamin B_12_ yield produced by yeast strains [45]. These findings highlight the critical role of inoculum size in optimizing vitamin B_12_ production.

**Table 3:** Effect of inoculum size on the biomass and vitamin B_12_ amount produced by four *Bacillus* strains

**Strain**	5% inoculum			10% inoculum			15% inoculum		
	**Biomass g/100 mL**	**Vitamin B**_**12**_**µg/100 mL culture**	**Vitamin B**_**12**_**µg g**^**−1**^**cells**	**Biomass g/100 mL**	**Vitamin B**_**12**_**µg/100 mL culture**	**Vitamin B**_**12**_**µg g**^**−1**^**cells**	**Biomass g/100 mL**	**Vitamin B**_**12**_**µg/100 mL culture**	**Vitamin B**_**12**_**µg g**^**−1**^**cells**
**MZ01**	0.5 ± 0.41	10.03 ± 0.01**	18.81	0.9 ± 0.58	174.14 ± 0.11*	193.49	0.91 ± 0.41	29.88 ± 0.10**	32.85
**MZ08**	0.935 ± 0.08	1.45 ± 0.00	1.55	0.4 ± 0.02	174.14 ± 0.13	435.35	1.1 ± 0.00	2.67 ± 0.01	2.43
**JT17**	0.1 ± 0.16	8.93 ± 0.00	89.28	0.1 ± 0.00	10.60 ± 0.03	106	1.01 ± 0.01	17.88 ± 0.05	17.71

Production conditions:12% glucose as a carbon source, at 30°C and 110 rpm. Data are means of three replicate values ± standard error of the mean

*P < 0.05

**P < 0.01

***P < 0.001

### Optimization of vitamin B_12_ production by statistical modeling using response surface methodology (RSM)

The Plackett–Burman design was employed to identify the optimal conditions for vitamin B_12_ production by the *Peribacillus acanthi* MZ01 strain, considering the impact of parameter interactions. Parameters such as inoculum size (10%), temperature, pH, shaking rate, fermentation time, salt concentration, and glucose levels were investigated for optimization using RSM. Experimental results regarding vitamin B_12_ concentration (µg/100 mL) and corresponding parameter settings are shown in Table S5. The highest concentration of vitamin B_12_ (18.21 µg/100 mL) was achieved under conditions where temperature, pH, shaking rate, fermentation time, salt concentration, and glucose levels were set at 35ºC, 7.5, 200 rpm, 24 h, 0%, and 18%, respectively. Conversely, the lowest concentration of vitamin B_12_ (0.22 µg/100 mL) was observed at 30ºC, 6.5 pH, 100 rpm, 60 h fermentation time, 1.8 salt concentration, and 12% glucose levels. Furthermore, low levels of vitamin B_12_ were also observed under conditions where temperature was set at 30ºC, pH at 7.5, shaking rate at 200 rpm, fermentation time of 60 h, salt concentration of 0%, and glucose levels at 18%.

Table S6 shows the ANNOVA analysis of response-surface Quadratic regression model where the F test and the corresponding *p*-values were estimated. The coefficient of determination (R2) for the following regression equation was 0.9648 (Table S7), indicating the adequacy of the model to accurately predict the response, as shown in Figure S1. The regression equation utilized to predict the Vitamin B_12_ concentration is as follows:

$$\mathrm{Vitamin}\;{\mathrm B}_{12}\;\mathrm{concentration}\;=\;3.498\;+\;0.475\mathrm A\;+\;0.272\mathrm B\;\;+\;0.489\mathrm C\;+0.629\mathrm D\;+\;0.582\mathrm E\;+\;0.545\mathrm F\;-\;0.046\mathrm{AB}\;-0.094\mathrm{AC}\;+\;0.237\mathrm{AD}\;-\;0.353\mathrm{AF}\;+\;0.185\mathrm{BC}\;+\;0.174\mathrm{BD}\;-\;0.110\mathrm{BE}\;-\;0.135\mathrm{CE}\;-\;0.213\mathrm{DF}\;+\;{1.190\mathrm A}^2$$

These findings are in line with a previous study by Mohammed et al. (2014) on *Bacillus megaterium*, where optimization conditions for vitamin B_12_ production were enhanced using Design Expert software [38]. The employed models were significant (*P* < 0.05), with coefficients of determination (R2) exceeding 90%, suggesting an accurate representation of the relationship between response and independent variables.

Figure S2 illustrates the impact of three values (-1, 0, and 1) per parameter on vitamin B_12_ yield. Optimal conditions for vitamin B_12_ production for each factor were determined as follows: temperature (35ºC), pH (7.5), shaking rate (200 rpm), glucose concentration (18%), salt concentration (4%), and fermentation time (24 h).

**Fig. 3:**
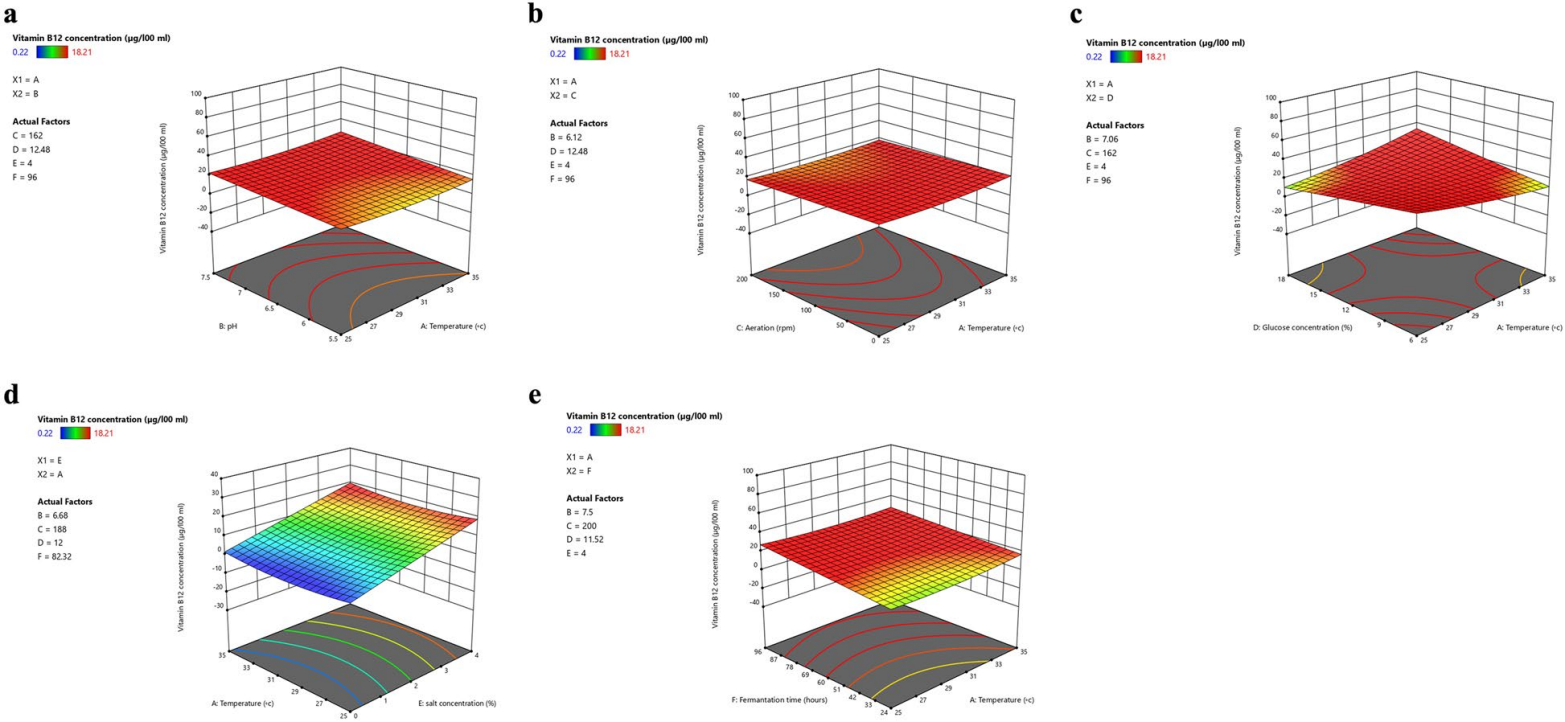
The 3D response surface and contour plots of the two-factor interaction effects on the yield of vitamin B_12_ production. **a** The interaction between temperature and pH. **b** The interaction between temperature and aeration. **c.** The interaction between temperature and glucose concentration. **d** The interaction between temperature and salt concentration. **e** The interaction between temperature and fermentation time

The 3D surface plots in Fig. 3 show the two-variable interaction effects among various parameters on vitamin B_12_ yield. Accordingly, an increase in vitamin B_12_ yield is observed when temperature increased from 30ºC to 35ºC and pH increased from 7 to 7.5, while maintaining shaking rate, glucose concentration, salt concentration, and fermentation time at 162 rpm, 14.88%, 4%, and 46.32 h, respectively (Fig. 3a). Similarly, Fig. 3b demonstrates enhanced vitamin B_12_ amount when temperature increased from 30ºC to 35ºC and shaking rate increased from 150 to 200 rpm, while maintaining pH, glucose concentration, salt concentration, and fermentation time at 6.12, 12.48%, 4%, and 96 h. Figure 3c, d, e display similar trends, showing that specific interactions of glucose concentration (higher than 9%), salt concentration (higher than 2%), and fermentation time (higher than 60 h) with temperature higher than 30ºC, while maintaining other parameters at optimal levels resulted in increased vitamin B_12_ amount. Notably, there is also a significant interaction between BD (pH and glucose concentration) and DF (glucose concentration and fermentation time) (Table S6). These significant interactions further emphasize how multiple factors synergistically influence the strain’s response for vitamin B_12_ production. For instance, maintaining an optimal pH alongside the glucose concentration ensures that enzymatic and metabolic functions are sustained, while also protecting the strain from the adverse effects of pH fluctuations on protein stability. The composition of the growth media, particularly the inclusion of glucose, has been shown to play a crucial role in maintaining pH stability, which, in turn, significantly influences vitamin B_12_ production. Glucose serves not only as a primary carbon source but also affects the metabolic pathways of the strain, leading to shifts in pH during fermentation [46]. The glucose concentration and fermentation time (DF interaction) are critical for energy supply and biosynthesis, directly impacting cell growth and vitamin B_12_ production. Wang et al., 2014 revealed that vitamin B_12_ production has been increased at a fermentation time of 96 h, and the consumption rate of glucose is directly related to the fermentation time affecting the vitamin B_12_ yield [47].

### Production of vitamin B_12_ using molasses synthetic medium by MZ01 strain

**Fig. 4:**
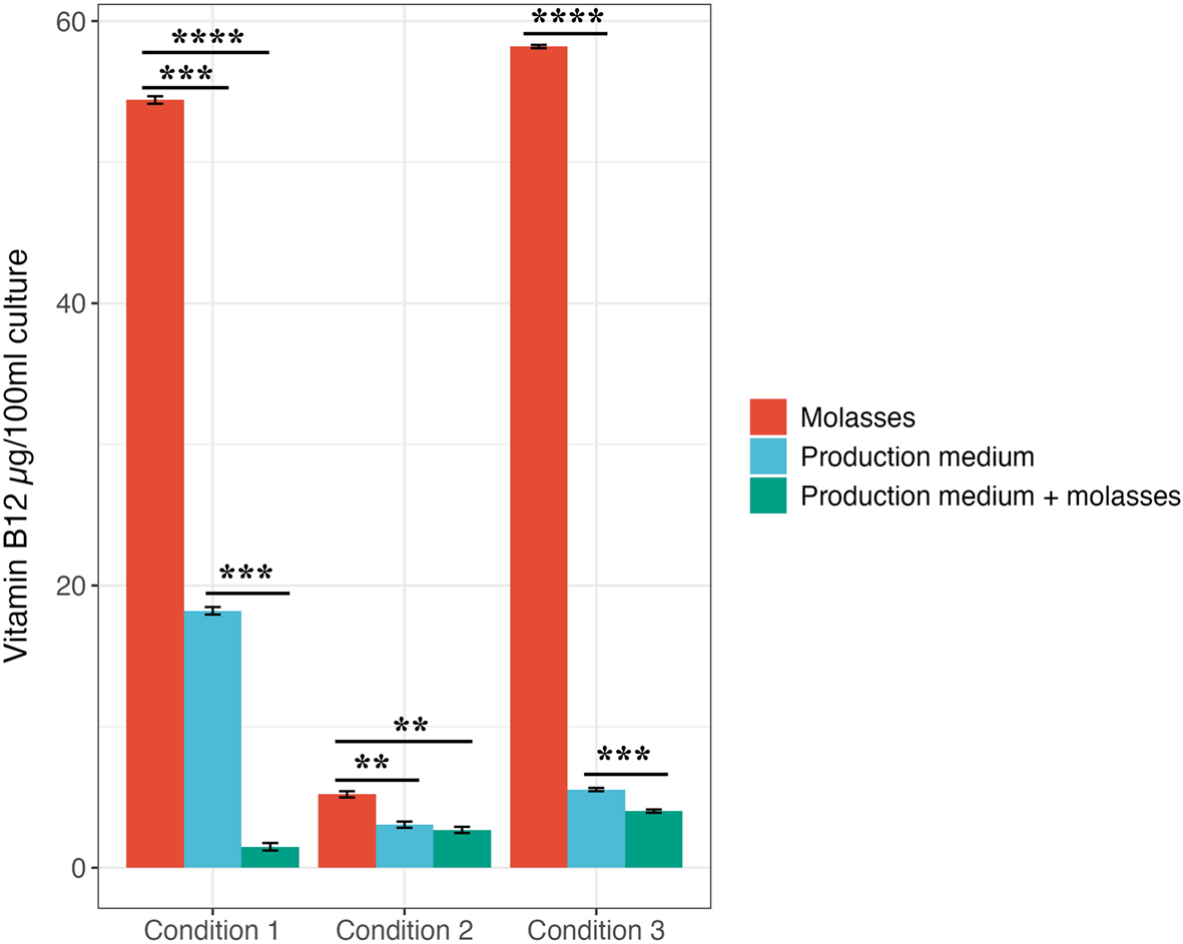
Vitamin B_12_ production using molasses as a production medium compared to molasses only and artificial production media containing glucose as a carbon source under three different production conditions. Data represent the mean of three replicates. Error bars represent the standard error of the mean. *P < 0.05. **P < 0.01, ***P < 0.001. ****P < 0.0001

Figure 4 and Table S8 demonstrate a significant increase in vitamin B_12_ production when using molasses alone compared to either synthetic molasses medium or a standard production medium. Three production conductions with different factor combinations were selected from the actual and predicted results of RSM to optimize vitamin B_12_ production by MZ01 strain.

Notably, the highest yields of vitamin B_12_ were achieved under conditions 3 and 1 using molasses as a production medium, particularly observed in RSM run 26, with values of 58.19 µg/100 mL and 54.4 µg/100 mL, respectively. While the lowest yield of 5.2 µg/100 mL was obtained under condition 2.

When comparing the yield of vitamin B_12_ from run 26, condition 1, and condition 3 using a production medium with glucose (18.21 µg/100 mL and 5.54 µg/100 mL), it was observed that this yield was reduced to 1.49 and 4.01 µg/100 mL, respectively, when utilizing synthetic molasses medium. This reduction in yield could potentially be attributed to the interference of molasses with other components in the production medium [15, 48]. Molasses alone can provide the necessary nutrients required for vitamin B_12_ production by the strain, but when mixed with synthetic medium, the interaction between molasses and the synthetic components might result in interference, leading to lower productivity. The balance and composition of nutrients in molasses alone may be more favorable for the strain, while the combined medium may introduce complexities that hinder optimal production.

Overall, molasses exhibits promising potential as a valuable by-product for augmenting vitamin B_12_ production, contingent upon management of production-influencing factors.

## Conclusion

In this study, various *Bacillus* strains were isolated and identified as vitamin B_12_ producers, with *Peribacillus acanthi* MZ01 being characterized for the first time as a potent vitamin B_12_ producer. The genetic circuits responsible for vitamin B_12_ biosynthesis were identified in the MZ01 strain, confirming its capability to produce this essential vitamin. The optimization of production conditions using the OFAT method and RSM identified the optimal conditions for vitamin B_12_ production and provided valuable insights into the interactions between different growth factors. Additionally, the use of molasses as a carbon source enhanced vitamin B_12_ production compared to glucose, suggesting that molasses could serve as an effective and economical substrate for industrial-scale production. This finding highlights the potential of using *Peribacillus acanthi* MZ01 strain for high-yield vitamin B_12_ production. Further research is required to scale up the production to achieve commercial viability for large-scale vitamin B_12_ production.

## Supplementary Information

Supplementary Material 1.

Supplementary Material 2.

## Data Availability

Sequence data that support the findings of this study have been deposited in the NCBI database with the primary accession code PP301893 Data is provided within the manuscript or supplementary information files.
