# Supplementary material for: Enhanced vitamin B12 production by isolated Bacillus strains with the application of response surface methodology
Source: BMC Biotechnol. 2024 Nov 12;24:90. doi: 10.1186/s12896-024-00919-5 (PMC11555979; doi:10.1186/s12896-024-00919-5)
Supplement: Supplementary file 1 — Supplementary Material 1. [file 12896_2024_919_MOESM1_ESM.docx]

**Table S1** Optimized production conditions for vitamin B_12_ using molasses as a production medium (Conditions 1, 2, and 3)

| **Experiment** | **Temperature (ºC)** | **Fermentation time (hours)** | **Salt concentration %** | **pH** | **Glucose concentration %** | **Aeration (rpm)** |
| --- | --- | --- | --- | --- | --- | --- |
| **condition 1** | 35 | 24 | 0 | 7.5 | 18 | 200 |
| **condition 2** | 29.2 | 91.67 | 3.16 | 5.75 | 10.21 | 197.95 |
| **condition 3** | 25.2 | 85.3 | 1.4 | 5.6 | 7.9 | 149.5 |

**Table S2** Cellular morphologies and number of vitamin B_12_-producing microorganisms isolates

| **microbial groups** | **Total number of isolates** | **number of vitamin B_12_-producing microorganisms** |
| --- | --- | --- |
| **Bacilli** | 39 | 9 |
| **Cocci** | 21 | 0 |
| **Actinomycetes** | 5 | 0 |
| **Yeast** | 22 | 6 |
| **Total** | 87 | 15 |

**Table S3** Identification of vitamin B_12_ producing isolates by MALDI-TOF MS technique

| **Isolate Code** | **Yeast or Bacterial Identification** | **Similarity %**  **Confidence %** |
| --- | --- | --- |
| **MZ01** | *Bacillus species* | 99.38 |
| **MZ08** | *Bacillus subtilis* | 99.23 |
| **CB09** | *Bacillus species* | 99.34 |
| **JT10** | *Bacillus subtilis* | 99.04 |
| **BY11** | *Bacillus subtilis* | 98.79 |
| **JT17** | *Bacillus subtilis* | 98.07 |

**Table S4** Pathogenicity of the identified strains as indicated by the hemolysis test

| **Strian Code** | Strains | Hemolysis results |
| --- | --- | --- |
| **MZ01** | *Peribacillus acanthi* | γ hemolysis (-) |
| **MZ08** | *Bacillus subtilis* | γ hemolysis (-) |
| **CB09** | *Bacillus species* | β hemolysis (+) |
| **JT10** | *Bacillus subtilis* | γ hemolysis (-) |
| **BY11** | *Bacillus subtilis* | γ hemolysis (-) |
| **JT17** | *Bacillus subtilis* | γ hemolysis (-) |

**Table S5** The experimental design matrix of the variables involved in the modeling using RSM, and the obtained responses represented as biomass and vitamin B_12_ yield produced via MZ01 strain under different conditions

| **Run** | **Std** | **A** | **B** | **C** | **D** | **E** | **F** | **Biomass g/100 mL** | **Vitamin B_12_ µg/100 mL culture** | **Vitamin B_12_ µg g^-1^cells** |
| --- | --- | --- | --- | --- | --- | --- | --- | --- | --- | --- |
| **1** | 18 | 30.5 | 60 | 2 | 6.5 | 12 | 100 | 0.20 | 2.97 | 14.85 |
| **2** | 26 | 30 | 60 | 2.2 | 6.5 | 12 | 100 | 0.13 | 2.3 | 17.69 |
| **3** | 4 | 35 | 96 | 4 | 5.5 | 6 | 200 | 0.53 | 4.31 | 8.13 |
| **4** | 20 | 30 | 60 | 2 | 6.6 | 12 | 100 | 0.13 | 0.29 | 2.23 |
| **5** | 2 | 35 | 24 | 4 | 7.5 | 6 | 200 | 0.93 | 3.15 | 3.39 |
| **6** | 11 | 25 | 96 | 0 | 7.5 | 6 | 0 | 1.06 | 0.74 | 0.69 |
| **7** | 19 | 30 | 60 | 2 | 6.4 | 12 | 100 | 0.40 | 1.041 | 2.60 |
| **8** | 22 | 30 | 60 | 2 | 6.5 | 12 | 110 | 0.53 | 2.97 | 5.60 |
| **9** | 21 | 30 | 60 | 2 | 6.5 | 12 | 90 | 0.07 | 0.22 | 3.14 |
| **10** | 5 | 25 | 96 | 4 | 7.5 | 18 | 0 | 0.47 | 0.59 | 1.25 |
| **11** | 28 | 30 | 63.6 | 2 | 6.5 | 12 | 100 | 0.13 | 1.78 | 13.69 |
| **12** | 17 | 29.5 | 60 | 2 | 6.5 | 12 | 100 | 0.13 | 0.89 | 6.85 |
| **13** | 27 | 30 | 56.4 | 2 | 6.5 | 12 | 100 | 0.47 | 0.52 | 1.11 |
| **14** | 8 | 35 | 96 | 4 | 7.5 | 6 | 0 | 0.13 | 0.33 | 2.54 |
| **15** | 14 | 25 | 96 | 4 | 5.5 | 18 | 200 | 1.20 | 2.15 | 1.79 |
| **16** | 24 | 30 | 60 | 2 | 6.5 | 12.6 | 100 | 0.33 | 0.67 | 2.03 |
| **17** | 23 | 30 | 60 | 2 | 6.5 | 11.4 | 100 | 0.13 | 0.52 | 4.00 |
| **18** | 25 | 30 | 60 | 1.8 | 6.5 | 12 | 100 | 0.13 | 0.22 | 1.69 |
| **19** | 16 | 25 | 24 | 0 | 5.5 | 6 | 0 | 0.60 | 0.44 | 0.73 |
| **20** | 13 | 25 | 24 | 4 | 5.5 | 18 | 0 | 0.33 | 3.49 | 10.57 |
| **21** | 7 | 25 | 24 | 0 | 7.5 | 6 | 200 | 0.40 | 1.93 | 4.82 |
| **22** | 9 | 35 | 24 | 0 | 5.5 | 18 | 0 | 0.73 | 3.27 | 4.48 |
| **23** | 29 | 30 | 60 | 2 | 6.5 | 12 | 100 | 0.07 | 0.67 | 9.57 |
| **24** | 6 | 35 | 96 | 0 | 5.5 | 18 | 200 | 0.73 | 2.08 | 2.85 |
| **25** | 12 | 35 | 24 | 4 | 5.5 | 6 | 0 | 0.26 | 3.05 | 11.73 |
| **26** | 1 | 35 | 24 | 0 | 7.5 | 18 | 200 | 1.53 | 18.21 | 11.9 |
| **27** | 15 | 25 | 24 | 4 | 7.5 | 18 | 200 | 0.40 | 4.38 | 10.95 |
| **28** | 10 | 25 | 96 | 0 | 5.5 | 6 | 200 | 0.87 | 8.81 | 10.13 |
| **29** | 3 | 30 | 96 | 0 | 7.5 | 18 | 0 | 0.47 | 0.67 | 1.42 |
| **Symbol** | | **Factor** | | | | | |  | **Levels** |  |
|  |  |  |  |  |  |  |  | **-1** | **0** | **1** |
| **A** | | Temperature (ºC) | | | | | | 25 | 30 | 35 |
| **B** | | Fermentation time (hours) | | | | | | 24 | 60 | 96 |
| **C** | | Salt concentration % | | | | | | 0 | 2 | 4 |
| **D** | | pH | | | | | | 5.5 | 6.5 | 7.5 |
| **E** | | Glucose concentration % | | | | | | 6 | 12 | 18 |
| **F** | | Aeration (rpm) | | | | | | 0 (static) | 100 | 200 |

**Table S6** Statistical analysis of variance (ANOVA) of Placket-Burman design for all parameters affecting the vitamin B_12_ production using MZ01 strain

| **Source** | **Sum of Squares** | **df** | **Mean Square** | **F-value** | **p-value** |  |
| --- | --- | --- | --- | --- | --- | --- |
| **Model** | 338.28 | 16 | 21.14 | 20.56 | < 0.0001 | significant |
| **A-Temperature** | 8.62 | 1 | 8.62 | 8.38 | 0.0135 |  |
| **B-pH** | 0.1096 | 1 | 0.1096 | 0.1066 | 0.7497 |  |
| **C-Aeration** | 62.79 | 1 | 62.79 | 61.07 | < 0.0001 |  |
| **D-Glucose concentration** | 10.7 | 1 | 10.7 | 10.41 | 0.0073 |  |
| **E-salt concentration** | 2.16 | 1 | 2.16 | 2.1 | 0.1725 |  |
| **F-Fermantation time** | 0.7938 | 1 | 0.7938 | 0.7721 | 0.3968 |  |
| **AB** | 15.8 | 1 | 15.8 | 15.37 | 0.002 |  |
| **AC** | 3.44 | 1 | 3.44 | 3.35 | 0.0923 |  |
| **AD** | 2.59 | 1 | 2.59 | 2.52 | 0.1386 |  |
| **AF** | 28.46 | 1 | 28.46 | 27.68 | 0.0002 |  |
| **BC** | 1.09 | 1 | 1.09 | 1.06 | 0.3241 |  |
| **BD** | 36.97 | 1 | 36.97 | 35.95 | < 0.0001 |  |
| **BE** | 9.77 | 1 | 9.77 | 9.5 | 0.0095 |  |
| **CE** | 25.96 | 1 | 25.96 | 25.25 | 0.0003 |  |
| **DF** | 58.06 | 1 | 58.06 | 56.47 | < 0.0001 |  |
| **A²** | 40.66 | 1 | 40.66 | 39.55 | < 0.0001 |  |
| **Residual** | 12.34 | 12 | 1.03 |  |  |  |
| **Cor Total** | 350.62 | 28 |  |  |  |  |
| **Lack of fit** |  |  |  | 0 |  | Not significant |

**Table S7** Coefficient of Placket-Burman design for all parameters affecting the vitamin B_12_ production using MZ01 strain

| **Factor** | **Coefficient Estimate** | **df** | **Standard Error** | **95% CI Low** | **95% CI High** | **VIF** |
| --- | --- | --- | --- | --- | --- | --- |
| **Intercept** | 1.15 | 1 | 0.2817 | 0.54 | 1.77 |  |
| **A-Temperature** | 0.7333 | 1 | 0.2533 | 0.1814 | 1.29 | 1 |
| **B-pH** | 0.0827 | 1 | 0.2533 | -0.4693 | 0.6347 | 1 |
| **C-Aeration** | 1.98 | 1 | 0.2533 | 1.43 | 2.53 | 1 |
| **D-Glucose concentration** | 0.8174 | 1 | 0.2533 | 0.2654 | 1.37 | 1 |
| **E-salt concentration** | 10.4 | 1 | 7.17 | -5.22 | 26.02 | 801 |
| **F-Fermantation time** | 6.3 | 1 | 7.17 | -9.32 | 21.92 | 801 |
| **AB** | 0.9938 | 1 | 0.2535 | 0.4414 | 1.55 | 1 |
| **AC** | 0.4637 | 1 | 0.2535 | -0.0886 | 1.02 | 1 |
| **AD** | 11.38 | 1 | 7.17 | -4.25 | 27.01 | 801 |
| **AF** | -1.33 | 1 | 0.2535 | -1.89 | -0.7814 | 1 |
| **BC** | 7.38 | 1 | 7.17 | -8.25 | 23.01 | 801 |
| **BD** | 1.52 | 1 | 0.2535 | 0.9677 | 2.07 | 1 |
| **BE** | -0.7813 | 1 | 0.2535 | -1.33 | -0.2289 | 1 |
| **CE** | -1.27 | 1 | 0.2535 | -1.83 | -0.7214 | 1 |
| **DF** | -1.91 | 1 | 0.2535 | -2.46 | -1.35 | 1 |
| **A²** | 2.38 | 1 | 0.3792 | 1.56 | 3.21 | 1 |

**Table S8** Vitamin B_12_ productivity by MZ01 strain under production conditions 1, 2, and 3

| **Sugar type** | **Production medium using glucose as a carbon source** | | | **Molasses synthetic medium** | | | **Molasses only** | | |
| --- | --- | --- | --- | --- | --- | --- | --- | --- | --- |
| **conditions** | **Biomass**  **g/100ml.** | **Vitamin B_12_**  **µg/100ml culture** | **Vitamin B_12_**  **µg g^-1^cells culture** | **Biomass**  **g/100ml.** | **Vitamin B_12_**  **µg/100ml culture** | **Vitamin B_12_**  **µg g^-1^cells culture** | **Biomass**  **g/100ml.** | **Vitamin B_12_**  **µg/100ml culture** | **Vitamin B_12_**  **µg g^-1^cells culture** |
| **Condition 1** | 1.53±0.15 | 18.21±0.00 | 11.90 | 0.67±0.02 | 1.49±0.00 | 2.22 | 0.13±0.01 | 54.4±0.00 | 418.46 |
| **Condition 2** | 0.27±0.13 | 3.05±0.00 | 11.29 | 1.73±0.01 | 2.68±0.00 | 1.55 | 0.60±0.12 | 5.2±0.00 | 8.67 |
| **Condition 3** | 0.50±0.07 | 5.54±0.00 | 11.08 | 0.80±0.06 | 4.01±0.00 | 5.01 | 0.13±0.03 | 58.19±0.00 | 447.61 |

**Temperature (ºC):** Condition 1 (35), Condition 2 (29.2), Condition 3 (25.2).

**Fermentation time (hours):** Run 26 (24), Condition 2 (91.67), and Condition 3 (85.3).

**Salt concentration %:** Condition 1 (0), Condition 2 (3.16), Condition 3 (1.4).

**pH:** Run 26 (7.5), Predicted (5.75), Confirmation (5.6).

**Glucose concentration %:** Condition 1 (18), Condition 2 (10.21), Condition 3 (7.9).

**Aeration (rpm):** Condition 1 (200), Condition 2 (197.95), Condition 3 (149.5).


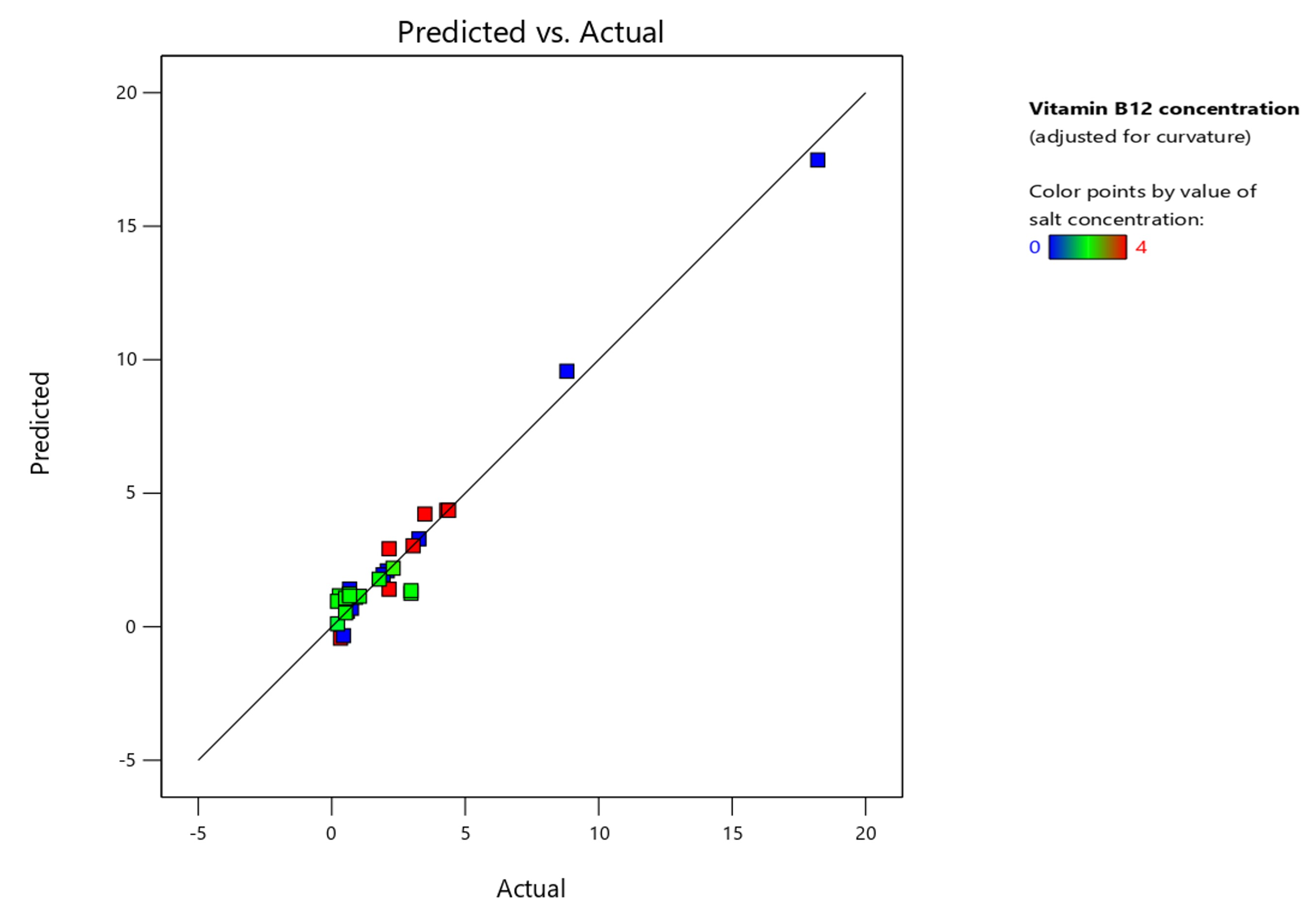


**Fig. S1** The difference between actual and predicted values. The color scale represents the concentration of vitamin B_12_.


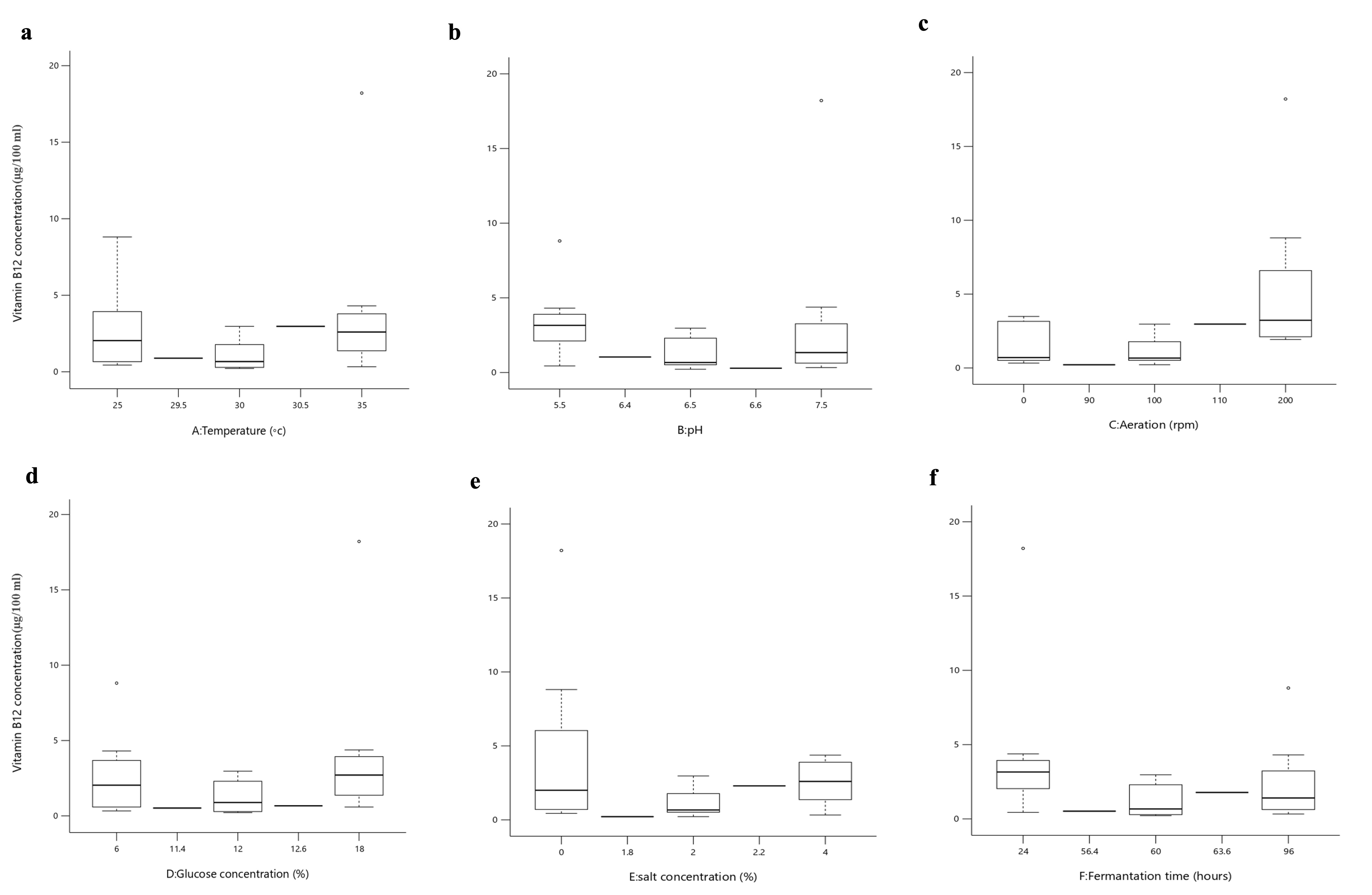


**Fig. S2** Box and whisker plots representing the mean for the effect of each single parameter on the vitamin B_12_ yield. **a.** The response effect of temperature**. b.** The response effect of pH. **c.** The response effect of aeration**. d.** The response effect of glucose concentration. **e.** The response effect of salt concentration**. f.** The response effect of fermentation time.
