# Supplementary material for: Enhanced vitamin B12 production by isolated Bacillus strains with the application of response surface methodology
Source: BMC Biotechnol. 2024 Nov 12;24:90. doi: 10.1186/s12896-024-00919-5 (PMC11555979; doi:10.1186/s12896-024-00919-5)
Supplement: Supplementary file 2 — Supplementary Material 2. [file 12896_2024_919_MOESM2_ESM.doc]

**Table 3** The effect of sugar type on the biomass and vitamin B_12_ amount produced by four *Bacillus* strains

| **Strain** | **Sucrose** | | | **Glucose** | | | **Fructose** | | | **Galactose** | | | **Lactose** | | |
| --- | --- | --- | --- | --- | --- | --- | --- | --- | --- | --- | --- | --- | --- | --- | --- |
|  | **Biomass**  **g/100ml** | **Vitamin B12**  **µg/100ml culture** | **Vitamin B12**  **µg g^-1^cells culture** | **Biomass**  **g/100ml** | **Vitamin B12**  **µg/100ml culture** | **Vitamin B12**  **µg g^-1^cells culture** | **Biomass**  **g/100ml** | **Vitamin B12**  **µg/100ml culture** | **Vitamin B12**  **µg g^-1^cells culture** | **Biomass**  **g/100ml** | **Vitamin B12**  **µg/100ml culture** | **Vitamin B12**  **µg g^-1^cells culture** | **Biomass**  **g/100ml** | **Vitamin B12**  **µg/100ml culture** | **Vitamin B12**  **µg g^-1^cells culture** |
| **MZ01** | 0.4± 0.07 | 5.13± 0.00 | 12.82 | 0.5± 0.07 | 10.03± 0.01 | 18.81 | 0.4±0.21 | 5.57± 0.00 | 11.94 | 0.4± 0.14 | 9.59± 0.01 | 23.97 | 0.3± 0.00 | 2.67± 0.01 | 10.03 |
| **MZ08** | 1.01± 0.01 | 3.46± 0.00 | 3.40 | 0.93± 0.01 | 1.45± 0.01 | 1.55 | 4.67± 0.15 | 2.59± 0.00 | 0.55 | 0.8± 0.07 | 2.56± 0.00 | 3.2 | 1.75± 0.01 | 4.24± 0.00 | 2.42 |
| **JT17** | 0.2± 0.07 | 6.02± 0.01 | 30.1 | 0.1± 0.00 | 8.93± 0.00 | 89.28 | 0.44± 0.02 | 15.27± 0.01 | 34.70 | 0.42± 0.01 | 9.69± 0.01 | 23.07 | 0.19± 0.01 | 6.8± 0.02 | 35.79 |

**Production conditions:**12% sugar type as a carbon source,

5% inoculum size, 30 ^o^C, and 110 rpm.
